# Near-IR emissive rare-earth nanoparticles for guided surgery

**DOI:** 10.7150/thno.40808

**Published:** 2020-02-03

**Authors:** Zhibei Qu, Jianlei Shen, Qian Li, Feng Xu, Fei Wang, Xueli Zhang, Chunhai Fan

**Affiliations:** 1 Joint Research Center for Precision Medicine, Shanghai Jiao Tong University & Affiliated Sixth People's Hospital South Campus, Southern Medical University Affiliated Fengxian Hospital, Shanghai 201499, China.; 2 School of Chemistry and Chemical Engineering, and Institute of Molecular Medicine, Renji Hospital, School of Medicine, Shanghai Jiao Tong University, Shanghai 200240, China.

**Keywords:** near infrared fluorescence, rare earth nanoparticle, bioimaging, image guided surgery

## Abstract

Intraoperative image-guided surgery (IGS) has attracted extensive research interests in determination of tumor margins from surrounding normal tissues. Introduction of near infrared (NIR) fluorophores into IGS could significantly improve the *in vivo* imaging quality thus benefit IGS. Among the reported NIR fluorophores, rare-earth nanoparticles exhibit unparalleled advantages in disease theranostics by taking advantages such as large Stokes shift, sharp emission spectra, and high chemical/photochemical stability. The recent advances in elements doping and morphologies controlling endow the rare-earth nanoparticles with intriguing optical properties, including emission span to NIR-II region and long life-time photoluminescence. Particularly, NIR emissive rare earth nanoparticles hold advantages in reduction of light scattering, photon absorption and autofluorescence, largely improve the performance of nanoparticles in biological and pre-clinical applications. In this review, we systematically compared the benefits of RE nanoparticles with other NIR probes, and summarized the recent advances of NIR emissive RE nanoparticles in bioimaging, photodynamic therapy, drug delivery and NIR fluorescent IGS. The future challenges and promises of NIR emissive RE nanoparticles for IGS were also discussed.

## 1. Introduction

Surgical operation is one of the most frequently used therapy to cancer treatment for centuries 1,2. In common cancer surgeries, intraoperative evaluation of margins of tumor is essential to determine the final curative result 3. However, it is mainly dependent on the visual senses and subjective palpation to decide excision 4 during the surgical operation. Inevitably, it is very difficult for the surgeons to discriminate the tumor margins from surrounding normal tissues 5,6. It has been reported that tumor recurrence happens as high as 20-30% after surgical therapy, and subsequent cancer metastasis largely increases the complexity 7,8. It is highly demanded to maximize tumor removal, minimize damage to the normal tissues and shorten surgical time 9. Thus, intraoperative image-guided surgery (IGS) 5,10,11 is introduced to provide real-time tumor visualization to oncological surgeons to do them a favor in cancer margin recognition^12^.

Among various optical imaging techniques 13, near-infrared (NIR) fluorescence imaging 14,15 is one of the latest trends in IGS applications^16^, for use in both fundamental medical research and clinical practice^17^,^18^. Due to advantages in reduction of light scattering, photon absorption and autofluorescence *via* broadening to the 700-1,700 nm NIR window 19, NIR fluorescence-based imaging technique provides high spatial resolution along with increased tissue penetration depths. Very recently, NIR phosphors that extended to the entire NIR window, including small molecules 20-22, inorganic nanoparticles 23,24, organic macromolecules 25,26 and quantum dots (QDs) 27,28 with tunable emission wavelength were developed 29. Besides the benefits of efficient detection of NIR photons, recently developed NIR fluorophores have enabled biomedical imaging 30 of specific biomarkers 31 and anatomical structures with better signal-to-noise ratio, application for preclinical animal studies 32,33, clinical diagnostics 34 and translational medicine 35.

Compared with the visible spectrum widely employed for fluorescence imaging, the studies over the broadly defined NIR window are still in their infancy 36. In the past decade, researches in NIR fluorescence imaging have focused on the conventional NIR window (NIR-I, 700-900 nm)^37^, and have recently extended their efforts to the second NIR window (NIR-II, 1,000-1,700 nm)^38^,^39^. The NIR-I window is typically named as the 'biological transparent window' because in this range there is low tissue absorption and fluorescence background *in vivo* (compared with the visible range)^40^. The studies of molecular imaging to the novel NIR-II window has been achieved by the development of biocompatible NIR fluorophores with increasingly longer wavelengths throughout the field of chemistry, materials science and nanotechnology 41. Also, we shall thank to the development of more efficient photon detectors with high NIR-II sensitivity as well as the drop of the price. It is more and more widely accepted that *in vivo* NIR-II fluorescence technology is superior to traditional NIR-I one due to the further reduced scattering, absorption and tissue autofluorescence 42.

**Figure 1:**
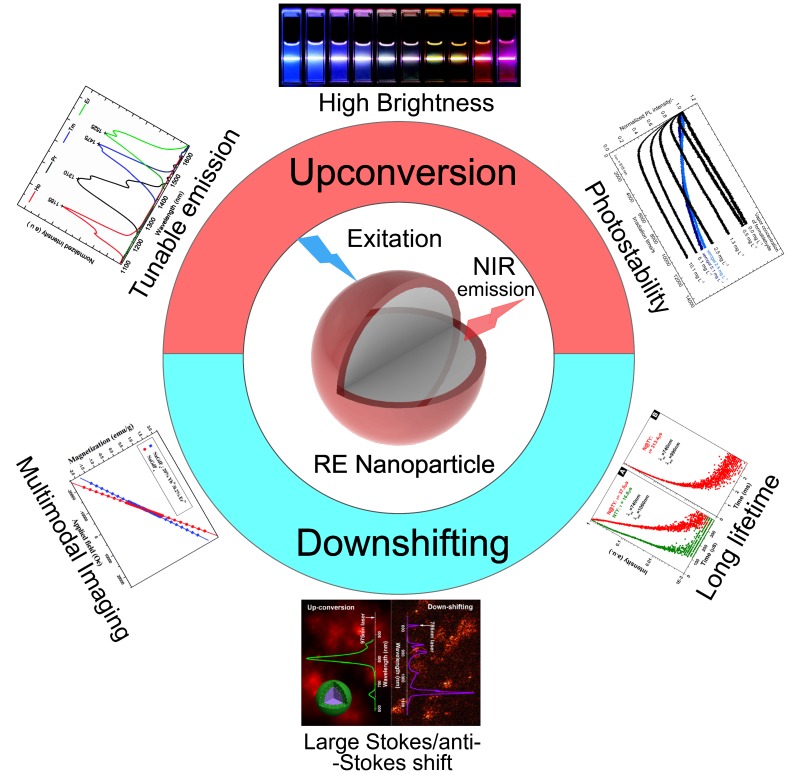
Characteristic properties of NIR emissive RE nanoparticles. Inset figures were adapted from Ref 63,69-72. Copyright 2014, Royal Society of Chemistry; Copyright 2008, 2011, 2013, 2016, American Chemical Society.

Among the existed NIR materials, Rare earth (RE) nanoparticles 43,44 can afford good stability, ease to fabricate 45,46, high emissive efficiency 47 and long luminescence lifetime to microseconds^48^. Compared with lanthanide chelates 49, QDs 50,51, polymers 52, and organic dyes 53,54, lanthanide-doped inorganic RE nanoparticles hold all the advantages, including tunable emission^55^, large Stokes shift 56, sharp emission peaks 57, and high chemical/photochemical stability 58 (Figure 1). Moreover, facile to multiple choices of doping 59, RE-doped inorganic materials can provide efficient emission from the ultraviolet (UV), passing through the whole visible range, to the mid-infrared region upon excitation 55,60,61. All the advantages mentioned above have enabled the promising potential of NIR emissive RE nanoparticles in bioimaging 62, theranostics 63,64, photothermal therapy 65,66, drug delivery 67, and also the clinical IGS 20,68.

In this review, we provided a comprehensive introduction to the RE based NIR emissive nanoparticles. We systematically compared the benefits and of RE nanoparticles with other NIR probes, and summarized the recent advances of NIR emissive RE nanoparticles in bioimaging, photodynamic therapy, drug delivery and NIR fluorescence enabled IGS. The future challenges and promises of RE nanoparticles with NIR emission were also discussed.

## 2. NIR emissive rare earth nanoparticles

### 2.1. Emission mechanism of RE nanoparticle

RE nanoparticles are important fluorescent materials, due to their ability to enable intriguing emission properties, including tunable fluorescence color (Figure 1) 70,73, long life-time photoluminescence 74, highly efficient upconversity 75, long persistent phenomenon 76,77. Generally speaking, RE elements are composed of 15 lanthanides (from lanthanum to lutetium), and usually plus scandium and yttrium. With abundant *f* shell orbitals, trivalent lanthanide (Ln) ions can exhibit sharp fluorescent emissions through intra-4*f* or 4*f*-5*d* transitions and thus are widely used as emitting centers in many fluorophores 78. There are multiple methods to endow RE nanoparticles with NIR emission, using either upconversion 79 or down-shifting mechanisms (Figure 1) 69, and even long lifetime fluorophore and “after-glow” persistent luminescence 80-82. Various luminescence features have been achieved in a wide spectrum of matrix materials, such as RE oxides, fluorides, and other matrices.

**Figure 2:**
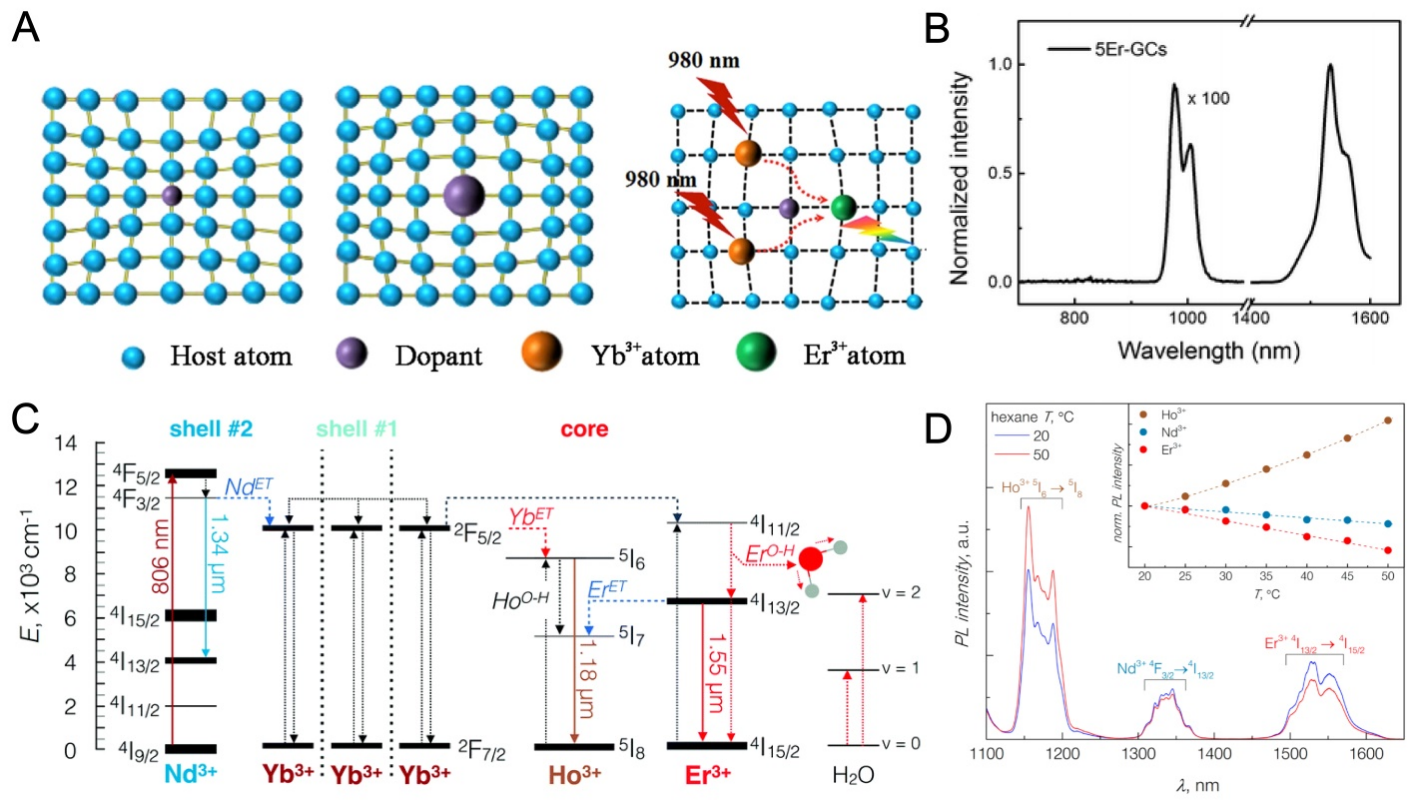
Luminescence mechanism of RE nanoparticles. (**A**) Scheme of emission mechanism of Yb, Er doped RE nanoparticles exited by 980 nm NIR light. Adapted with permission from 83, Copyright 2018, Nature Springer. (**B**) NIR-spectra of 5% Er^3+^ doped glass ceramics upon 400 nm irradiation. Adapted with permission from 84, Copyright 2015, Royal Society of Chemistry. (**C**) Principal operation scheme for the NIR-to-NIR emission of RE doped nanoparticles. (**D**) NIR emission bands of Ho^3+^ (1.18 µm), Nd^3+^ (1.34 µm), and Er^3+^ (1.55 µm) ions excited with 806 nm irradiation. Adapted with permission from 85, Copyright 2017, Royal Society of Chemistry.

Due to the involvement of multiple steps in one single luminescence process, including electron transition and the transition probability (Figure 2a), the excitation selection, multiphonon relaxation and energy transfer, Stokes shift and line broadening,^86^ the study of RE nanoparticle emission is highly confusing where lots of details remains unclear. For example, at least 6 states were involved in an Yb, Er, Nd-co-doped triple-layered core-shell NIR fluorophore 84. During the absorption process of 800 nm light, the ^4^I_9/2_→^4^F_5/2_ transition of Nd^3+^ is firstly involved. After that, the energy is fast transferred to the inner layer by a ^2^F_7/2_→^2^F_5/2_ process between Nd^3+^ and Yb^3+^. Consequent energy transfer happens by the co‐doped Yb^3+^ and then sensitize Er^3+^ (Yb^3+^→Er^3+^, ^4^I_15/2_→^4^I_11/2_). After all, the relaxation from the excited state of Er^3+^ finally releases a 1525 nm (^4^I_13/2_→^4^I_15/2_) photon *via* phonon vibration process (Figure 2b). Similarly, a complicated phonon-assisted Yb^3+^ (^2^F_5/2_) → Ho^3+^ (^5^I_6_) and Nd^3+^ (^4^F_3/2_) → Yb^3+^ (^2^F_5/2_) energy transfer mechanism was proposed in Yb^3+^, Ho^3+^, Nd^3+^ doped core/shell NaGdF_4_ nanoparticles and it was designed as a nanothermometer due to a temperature-dependent promotion of the electronic-to-vibrational energy transfer (Figure 2c,d). Steady/transient state fluorescence spectroscopy, fluorescence polarization spectroscopy, and femto-second laser pulse luminescence etc. were widely used to study the emission mechanisms of RE fluorophores 87,88. The better understanding of the luminescence mechanism will help to design better NIR probes as well as further broadened applications.

### 2.2. Material Subclasses of NIR emissive RE nanoparticles

**Figure 3:**
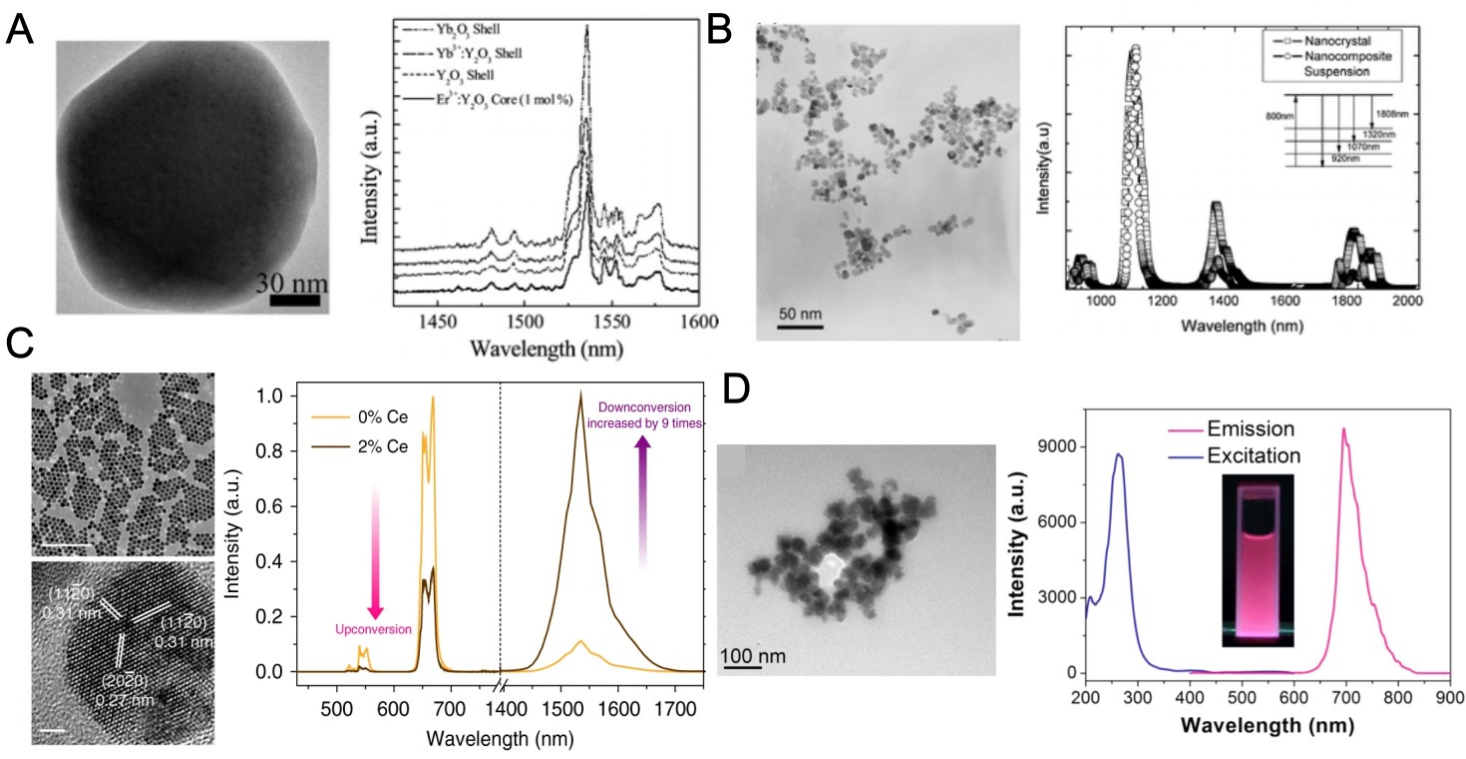
Subclasses of RE nanoparticles. TEM images (left) and NIR fluorescent spectra of (**A**) Yb_2_O_3_ nanoparticles, adapted with permission from 91, Copyright 2012, American Chemical Society. (**B**) YF_3_ nanoparticles, adapted with permission from 94, Copyright 2007, American Chemical Society. (**C**) NaYF_4_ nanoparticles, adapted with permission from 101, Copyright 2017, Nature Springer and (**D**) Zn_2.94_Ga_1.96_Ge_2_O_10_ nanoparticles, adapted with permission from 102, Copyright 2014, American Chemical Society.

RE oxides 89,90, in most cases, Y_2_O_3_, (Figure 3a) is the first generation NIR emissive lanthanide material 91,92. In a typical synthesis of RE oxides, the nanoparticles were synthesized through homogeneous precipitation and then high temperature calcination is required to increase the emission efficiency if necessary. In 2003, Vetrone and his coworkers investigated the upconversion emission of nanocrystalline and bulk Y_2_O_3_:Er^3+^ and the influence of the erbium concentration to the luminescence 93. They reported that by adjusting the doping concentration, a transition in emission from visible to NIR region was observed. Soga group further developed liposome encapsulated, Er-doped Y_2_O_3_ nanoparticles with various surface modifications as a fluorescent probe for NIR bioimaging 90. The authors introduced PEG on the liposome surface to avoid nonspecific interaction with proteins. Both microscopic and macroscopic NIR imaging systems were applied to image the organs of a mouse injected with the NIR-encapsulated liposomes as a demonstration of successful NIR bioimaging. But limited by the relatively low NIR emission efficiency of lanthanide oxides, their applications stop at organ bioimaging and no more clinical approaches were conducted.

RE fluorides 94,95, referring to YF_3_ and LaF_3_, 96 and maybe more frequently, NaYF_4_ and NaGdF_4_ 73,87, were the most widely used doping matrices (Figure 3c) for lanthanide phosphor 79. This is largely because NaLnF_4_ exhibits the lowest non-radiative energy loss and endow the highest quantum yield for a major of lanthanides doping. The NaLnF_4_ is still the most widely used matrix 97 for NIR emission through either upconversion or downshifting luminescence by different rare earth doping, largely on account to the high emission efficiency and extraordinary chemical stability. In addition, NaGdF_4_ also has excellent magnetic properties and it was widely employed as contrast agents in MRI 98,99. The classic synthesis of NaYF_4_ or NaGdF_4_ nanoparticles was conducted in oleic acid and 1‐octadecene through a solvothermal method, using lanthanide nitric salts or lanthanides acetates, reacting with NaF or NH_4_F. The versatile luminescent properties as well their intriguing magnetic and electronic properties of RE fluorides open an avenue to multi-mode molecular imaging and dual signal guided surgery 100. For example, Riman's group prepared highly NIR emissive fluoride nanopowders (LaF_3_: Nd and CaF_2_: Er) with solvothermal methods (Figure 3b). The quantum efficiencies were as high as 95% for LaF_3_: Nd and 51% for CaF_2_: Er, which are much higher than RE oxides 94. In 2013, Zhou *et al.* employed Tm and Nd doped NaGdF4 nanoparticles to efficient NIR-to-NIR upconversion and down-shifting emission, providing a dual mode platform for NIR fluorescence bioimaging and promisingly even magnetic resonance imaging (MRI) probes 69.

Recently, a series of new matrices were reported for NIR emission of lanthanides 80,103,104, where solid state high-temperature synthesis were usually employed. The new matrices bring new properties to NIR based bioimaging, such as long persistence emissive phenomenon and degradability in physiological fluids. For example, Scherman *et al.* reported to successfully prepare lanthanide doped Ca_0.2_Zn_0.9_Mg_0.9_Si_2_O_6_ nanoparticles with NIR persistent luminescence 82. The NIR persistent nanoparticles can be excited before injection to mouse, and the biodistribution of the nanoparticle can be monitored in real-time for more than 1 h without any external illumination source. The nanoparticles were modified with targeting ligands, would guide the nanoparticles specifically to lung, liver or to long-lasting blood circulation. This system can be employed to evaluate tumor mass and showed great clinical potential. Another similar work was reported by Yan's lab that they synthesized NIR emitting Zn_2.94_Ga_1.96_Ge_2_O_10_ nanoparticles co-doped with Cr^3+^, Pr^3+^ for long persistent luminescence (Figure 3d). The nanoprobe was further functionalized with gadolinium complexes and enabled a multimodal *in vivo* MRI and NIR luminescence imaging 102.

## 3. RE nanoparticles for NIR bioimaging

Fluorescence based bioimaging in the NIR window features deep tissue penetration, reduced tissue scattering, and decreased tissue autofluorescence. These advantages would largely improve the performance of nanoparticles in biological and pre-clinical applications. Hence, NIR fluorescent probes, especially RE nanoparticles, are constructed into platforms for NIR bioimaging 105, biosensing 106, drug delivery 107, photodynamic therapy and NIR based IGS 108. The application in bioimaging is the first step for successive preclinical studies and practices. The good performance in NIR bioimaging of RE nanoparticles plays as cornerstones for the follow-up photodynamic therapy, drug delivery and surgical navigation.

**Figure 4:**
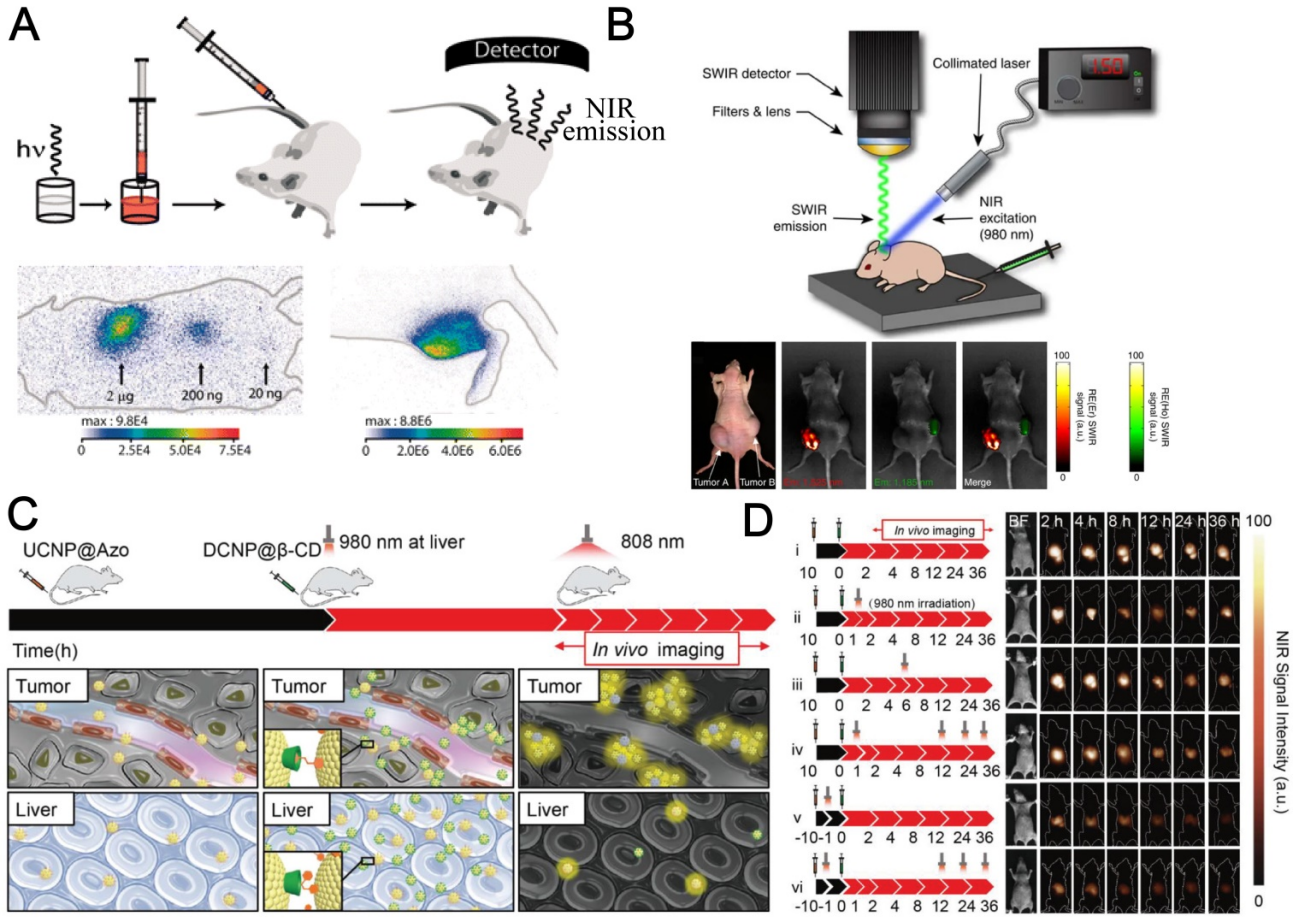
RE nanoprobes for NIR bioimaging *in vivo*. (**A**). Principal scheme for *in vivo* experiments and NIR bioimaging. NIR images of mice at different localizations with different nanoparticle amounts. Adapted with permission from 82, Copyright 2007, National Academy of Sciences. (**B**). Schematic of the portable short-wave infrared (SWIR) imaging prototype using 980 nm NIR excitation and the bioimaging for injected tumor on nude mouse. Adapted with permission from 114, Copyright 2013, Nature Springer. (**C**) Scheme illustration of assembly and NIR laser‐regulated disassembly of nanoprobes for stable and accurate NIR‐II bioimaging. (**D**) Schematic depiction of experimental timeline for the *in vivo* assembly and 980 nm laser‐triggered *in vivo* disassembly and NIR‐II fluorescence bioimaging results for the abdomen (1000 nm long‐pass filter) of the nude mice with murine epidermal tumor by two‐staged in‐sequence injection of RE nanoparticles (interval between two injections is 10 h) under 808 nm excitation. Adapted with permission from 115, Copyright 2018, John Wiley & Sons, Inc.

Various groups have successfully reported NIR emissive RE nanoparticles for bioimaging 109 (Figure 4a). Hammond's group constructed LbL-modified NIR-II nanoparticels from RE doped NaYF_4_ fluorescent materials to perform a side-by-side investigation and comparison for the biodistribution, pharmacokinetics, and toxicities of these probes 110. Moghe *et al.* reported a multispectral, real-time short-wavelength infrared imaging offering anatomical resolution using brightly emitting RE nanomaterials and demonstrate their practicability as a disease-targeted imaging method (Figure 4b) 111. RE nanomaterials modified with human serum albumin (HSA) endowed systemic study of biodistribution of the RE nanoparticles. It was reported by the authors that accumulation and retention in tumor tissue was improved after protein conjugation, which was visualized by the localized enhancement of NIR signal intensity (Figure 4b). The involvement of HSA was drawn as experiences by a lot of other studies and was verified to improve the biocapability and retention time in organs and tumors 112. Liu's group found another route of NIR emitting nanomaterials for theranostic applications on how RE moieties were involved. They fabricated a nanocomplex where Gd^3+^ chelate were functionalized onto HSA, conjugated with a NIR dye IR825 113. The albumin-based probe was capable of multimodal imaging and photothermal therapy (PTT). The authors also validated the practicability of an NIR 'photothermal ablation assisted surgery' strategy using the theranostic nanoassay, which is promising for future clinical cancer treatment.

In 2019, a surge of RE nanoparticles for NIR bioimaging have been reported by different research groups. Zhang's group reported that *in vivo* assembly and disassembly of supramolecularly engineered NIR‐II emissive RE nanoparticles (Figure 4 c,d) can greatly improve the quality of bioimaging 115. In another work by the same team, they succeeded in precise *in vivo* inflammation imaging technique using *in situ* responsive cross‐linking of glutathione‐modified NIR‐II lanthanide nanoparticles. NIR‐II signals in the inflamed area were observed within 10 min and lasted as long as 8 h. The signal-noise ratio of inflammatory bioimaging was enhanced 2.9‐fold compared with reference groups at the same time. Their ROS‐responsive *in vivo* crosslinking strategy provides a safe and easy route for the fast location of and long‐term imaging of inflamed areas 116. Li et al. proposed the poly(acrylic acid) (PAA)-modified NaLnF_4_:40Gd/20Yb/2Er nanorods (Ln = Y, Yb, Lu, PAA-Ln-NRs) with enhanced downshifting NIR-IIb emission for improved quality of bioimaging 117. The downshifting emission beyond 1500 nm is doubled by suppressing the upconversion path through Ce^3+^ doping. The explored bright NIR-IIb emitted PAA-Lu-NRs were used for a series of applications, including high sensitivity small tumor (∼4 mm) imaging, metastatic tiny tumor detection (∼3 mm), high spatial resolution (41 μm) tumor vessel visualization, and brain vessel imaging. Their findings opened the opportunity of utilizing the RE based NIR-IIb probe for *in vivo* tumor vessel/metastasis and noninvasive brain vascular imaging. It should be drawn more interests that Gu et al. reported an important progress of NIR bioimaging using RE nanoparticles 118. In their work, a time-domain (τ) based light transducer was applied instead of conventional spectra-domain signaling, serving as a new weapon for *in vivo* NIR imaging. The ytterbium-based transducer can convert the pulsed NIR irradiation into long-decaying luminescence with an efficiency approaching 100%. This technique can largely improve the signal-to-noise ratio and bioimaging quality in mice models.

DNA nanotechnology 119 also plays an important role in bioimaging using RE nanoparticles. DNA structures, including G‐quadruplexes 120, aptamers 121, molecular switches 122, framework nucleic acids 123 (FNAs, eg. DNA tetrahedrons 124,125), and origamis 126, were widely involved in design of probes for RE nanoparticles based bioimaging systems 127 or theranostic devices 128,129. In comparison of other materials such as inorganic gold nanoparticles 130,131, DNA nanostructures 132,133 showed extraordinary biocompatibility, degradability, low size dispersibility 134 and programmability 135. The reversible Watson-Crick pairing of DNA also provide a versatile platform to construct dynamic, programmable, precisely controlled devices 136 for sensing 137 and imaging in combination of RE nanoparticles. For example, Lu and his group introduced DNA modifications to RE nanoparticles and successfully obtained controllable assemblies of gold nanoparticles onto RE upconversion nanoparticles for improved drug delivery and bioimaging 138. Kuang* et al.* reported the self-assemblies of RE nanoparticles with DNA tetrahedrons and applied them as a chiral sensing platform for cell imaging and direct observation of autophagy 139.

## 4. RE nanoparticles for NIR photodynamic therapy (PDT) and drug delivery

The NIR bioimaging systems were widely studied in various biological applications and clinical attempts, such as cell and tissue imaging, tumour diagnosis and therapy, and surgical navigation. However, limited by the difficulties of clinical practices, most of the researches of NIR bioimaging did not reach the surgical guidance level. Considering this, we also concluded the recent progresses in photodynamic therapy (PDT) and drug delivery using rare earth nanoparticles since the requirements of the probes and the NIR imaging equipment are similar with IGS but practically much easier to achieve to a lot of research groups in this field. The highly related fields will share a view in material design, safety estimation, animal models and so on 140. For bioimaging and IGS applications, the performance is largely determined by the signal-to-background ratio and targeting affinity. It requires higher fluorescence efficiency, lower tissue photo-absorption and stabilized functionalization. Down-shifting RE nanoparticles with NIR-II emission excited by NIR-I laser is commonly used to achieve good *in vivo* bioimaging quality. For PDT and drug delivery design, higher photon energy is demanded to trigger the ROS generation or release of cargos. And upconversion nanoparticles that will give rise to the photon energies are preferred.

Photodynamic therapy (PDT) is a non-invasive treatment modality for a variety of diseases including cancer 141,142. A recent popular strategy to conduct PDT is based on a subclass of RE nanoparticles, upconversion nanoparticles (UCNPs). Upon NIR excitation, UCNPs emit visible light with anti-Stokes shifts, which can be applied to activate modified photosensitizers to produce reactive oxygen species (such as ^1^O_2_) and damage cancer cells through oxidative stress and activated metabolic autophagy 143. NIR-excited UCNPs can be utilized to activate photosensitizers in deep tissues and exhibit wider coverage of therapies and better efficiency than traditional PDT under visible or UV light illumination. Similarly, RE nanoparticles could also be used for NIR light-triggered drug release 144 through photothermal process or photochemical cascade reactions 145,146.

**Figure 5:**
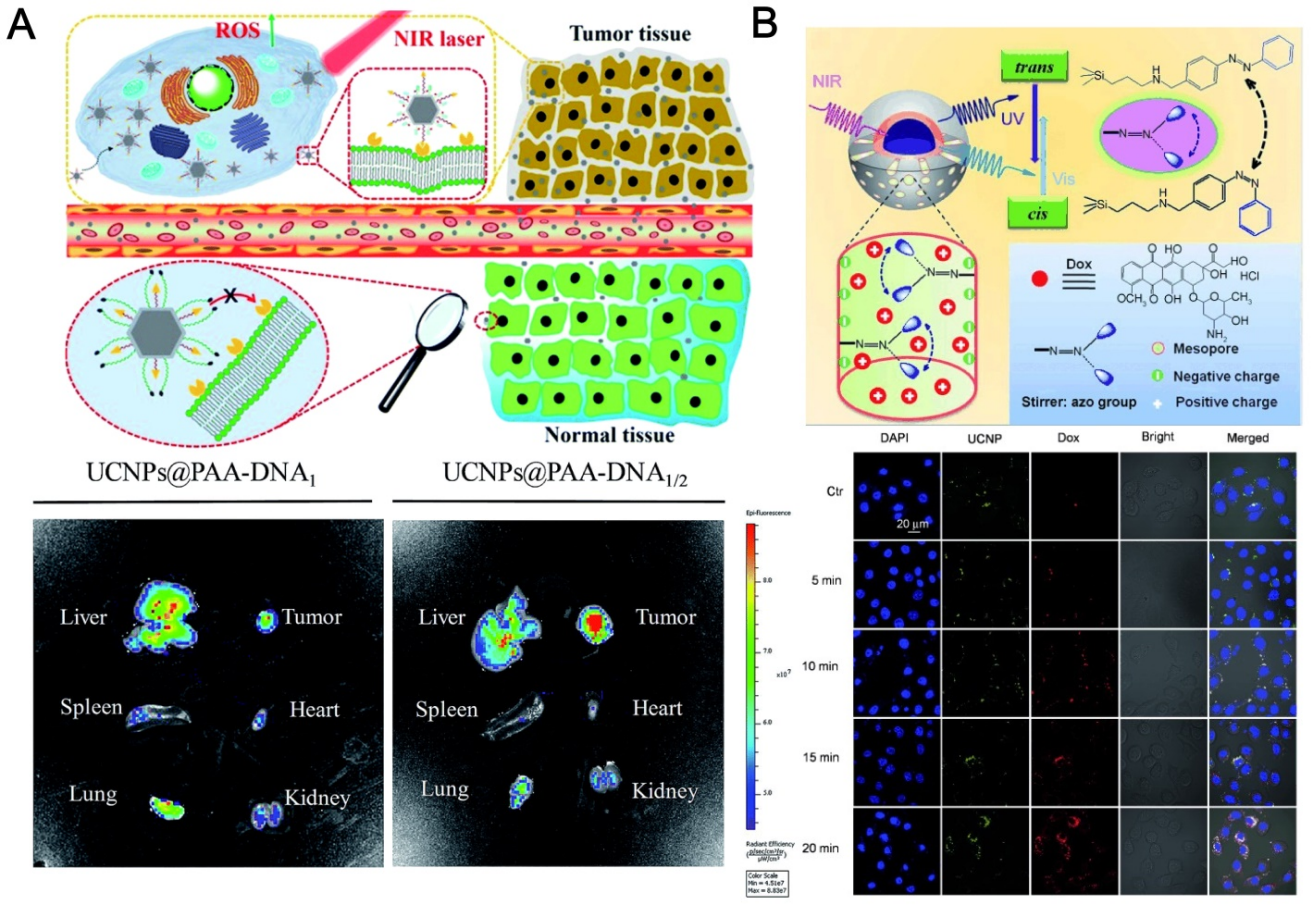
(**A**) Precise tumor targeting and specific PDT for cancer of UCNPs@PAA-DNA. *In vivo* imaging of five major organs harvested from a mouse at 8 h postinjection with UCNPs@PAA-DNA1(Ce6) (left) or UCNPs@PAA-DNA1/2 (right). Adapted with permission from 147, Copyright 2018, Royal Society of Chemistry. (**B**) Upper: NIR light‐triggered Dox release by making use of the upconversion property of UCNPs and trans-cis photoisomerization of azo molecules grafted in the mesopore network of a mesoporous silica layer. Down: CLSM observations of the photocontrolled Dox release in HeLa cells. Adapted with permission from 145, Copyright 2013, John Wiley & Sons, Inc.

The first *in vivo* UCNP-based PDT study on animals was demonstrated by Liu's team. 48 They applied non-covalently incorporated Ce6 onto PEGylated amphiphilic polymer-coated upconversion nanoparticles (UCNPs). The obtained UCNP-Ce6 complex could enter cancer cells and induce 4T1 cell death after being exposed to the 980-nm NIR light. The survival of mice after UCNP-Ce6 injection and PDT treatment was dramatically pro-longed compared to the control group. They also found that the injected UCNPs could be gradually cleared out after 2 months, determined by *ex vivo* ICP-AES measurement, without noticeable toxicity to the treated mice. It is valuable that the authors also compared the tissue penetration abilities for the same NIR probes induced by 980-nm NIR light and 660 nm visible light. It is observed that more singlet oxygens were generated under 660-nm illumination, in comparison to UCNP-Ce6 sample under the 980 nm excitation. But under 8 mm tissue (pork) blocking, 660 nm visible light will lose its power in singlet oxygen production but 980 nm NIR illumination remains high efficiency. Very recently, Yu et al. developed a pre-protective strategy using a switchable folic acid modified UCNPs conjugated with two types of DNA in different lengths. In normal tissues, folic acid is protected by longer DNA. The platform can be triggered in tumor site to exposed folic acids for tumor targeting and NIR PDT (Figure 5a) 147.

Besides the application of UCNPs in PDT, photo-responsive drug release systems using NIR triggering, have received remarkable emphasis in recent years, due to their promising potential in noninvasive theranostics at the site of nidus (*e.g.* tumors) 148. For example, Shi *et al.* fabricated mesoporous silica coated UCNPs modified by azobenzene molecules. 145 The anticancer drug doxorubicin (DOX) were controllably released from the outer layer of the mesoporous silica under NIR laser irradiation (Figure 5b). Qu *et al*. reported a NIR upconversion responsive system carrying two cargos (clioquinol and curcumin) to stepwise sequential release 149. When the UCNP platform is irradiated at low intensity of the NIR laser, clioquinol is first released for chelating with free metal ions such as Cu^2+^, which hinders the efficacy of curcumin. Subsequently, under higher intensity of NIR illumination, curcumin is subsequently released. The stepwise-release strategy can greatly improve the activity of curcumin for the inhibition of amyloid aggregation. Excess Cu^2+^ ions and superfluous ROS can be cleaned up by the NIR-triggered drug delivery platform.

## 5. Surgery guide using NIR emissive RE nanoparticles

Inspired by the success of bioimaging and PDT therapies using NIR emissive nanoparticles, researchers urged to put forwards the employment of NIR probes into clinical practices. Tian *et al.* used ZnGa_2_O_4_Cr_0.004_ (ZGC) nanoparticles for guided surgery during operation to accurate delineation of hepatocellular carcinoma (HCC) 23. ZGC showed excellent long-lasting NIR afterglow properties that lasted for hours, which can improve real-time guided surgical quality. Though the ZGC nanoparticles employed in this work were not consisting of any RE elements, the ZGC probes with NIR emission is surely a continuum of its prototype counterpart-- Zn_2.94_Ga_1.96_Ge_2_O_10_:Cr^3+^,Pr^3+^ nanoparticles, where RE element Pr plays as emitters 80.

**Figure 6:**
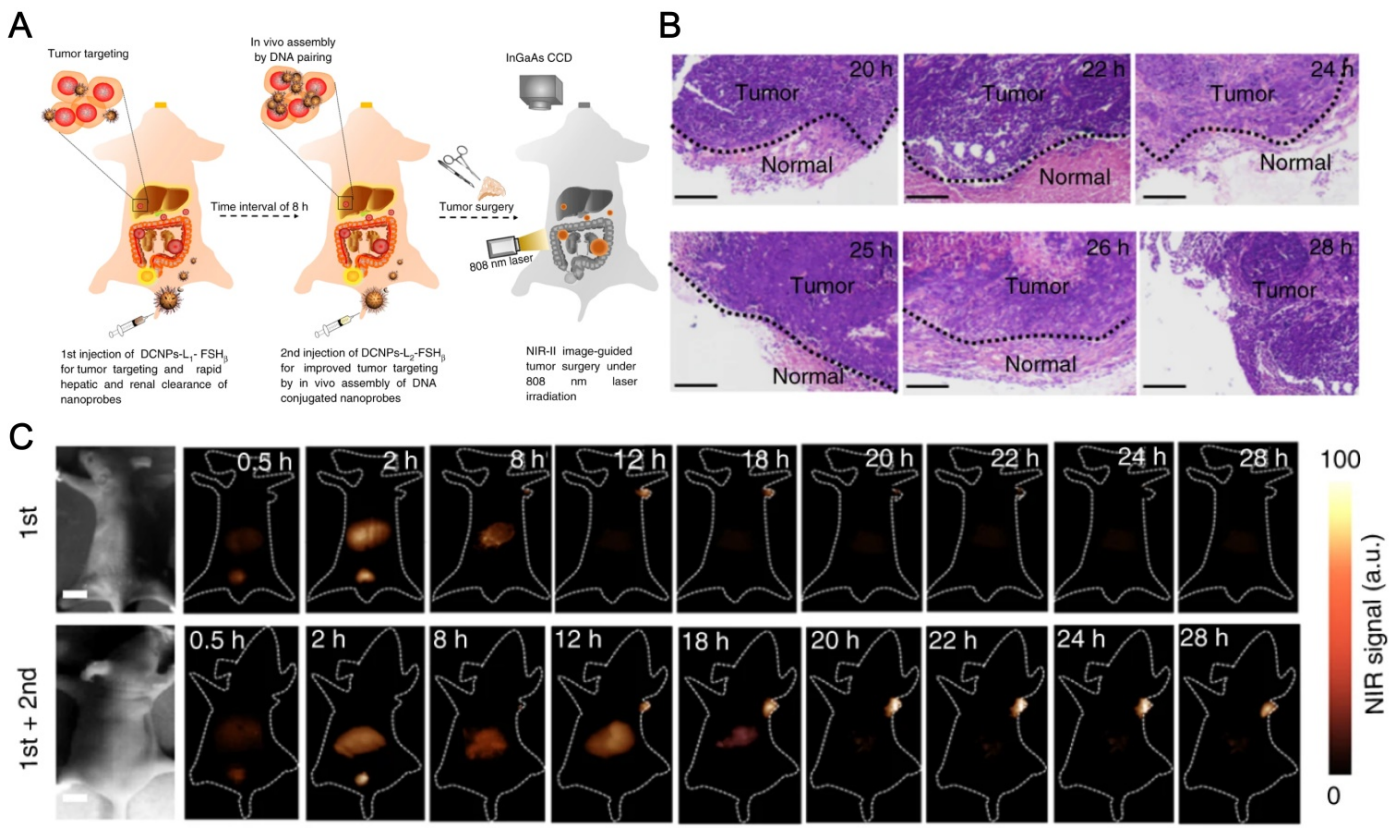
NaGdF_4_ based NIR-II nanoprobes *in-vivo* assembly to improve IGS for metastatic ovarian cancer. (**A**) Schematic illustration of NIR-II nanoprobes fabrication for ovarian metastasis surgery under NIR-II bioimaging guidance. (**B**) Hematoxylin and eosin (H&E) staining results of the tumors resected in 20-28 h PI under NIR-II fluorescence bioimaging guidance. (**C**) NIR-II fluorescence bioimaging (1000 nm long-pass filter) of the nude mice with murine epidermal tumor by single caudal vein first injection and two-staged in sequence injection (first + second) (interval between two injection is 8 h) under 808 nm excitation (fluence rate = 40 mW cm^-2^). The concentration of DCNPs in single injection is same to the sum of that for two-staged injection. All scale bars: 1 cm. Representative images are for n = 5 per group. Adapted with permission from 150, Copyright 2017, Nature Springer.

Very recently, Zhang's lab at Fudan University also reported *in vivo* assembly of the lanthanides doped NaGdF_4_ based NIR-II emitting nanoparticles to improve the IGS for metastatic ovarian cancer (Figure 6) 150. The NIR-II probes were modified with DNA and targeting peptides while the imaging quality is largely improved with good photostability and deep tissue penetration over 8 mm, in comparison to that of conventional organic dye, indocyanine green (ICG). The authors observed *in vivo* assembly of the nanoprobes, which increases the tumor retention period to 6 h, enabled precise tumor resection. Also, better tumor-to-normal tissue ratio is successfully achieved to facilitate the abdominal ovarian metastases surgical operation. The preclinical practice proved that metastases smaller than 1 mm can be completely excised under Zhang's NIR-II bioimaging guidance. This work is a milestone of the applications of RE based NIR emissive nanoparticles and greatly encourages researchers to bring NIR fluorescence IGS to clinical surgery.

**Figure 7:**
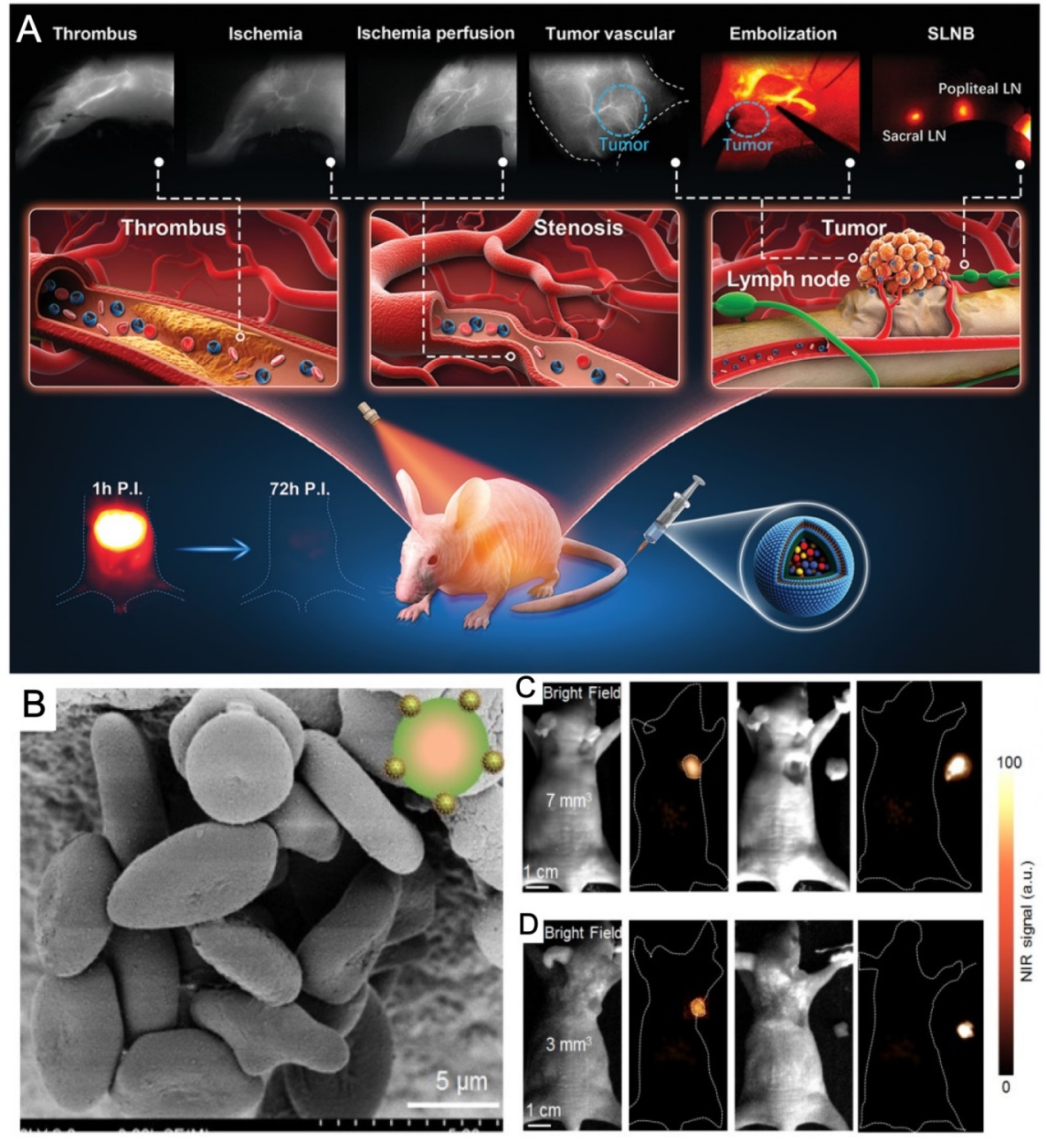
RE nanoparticles for surgical guidance with NIR imaging. (**A**) Schematic illustration of excretable RE nanoparticle for multifunctional biomedical imaging and ICG in the NIR-II window. (**B**) SEM images of multimodal probes enabled by red blood cell coated with NIR-II emissive lanthanide nanoparticles. Adapted with permission from 151, Copyright 2019, John Wiley & Sons, Inc. (**C, D**) NIR II fluorescence bioimaging results (12 h PI) of epidermal tumors with sizes of 7 mm^3^ (**C**) and 3 mm^3^ (**D**) and NIR II fluorescence bioimaging results after the surgical resection of tumors. Adapted with permission from 152, Copyright 2019, Ivyspring International Publisher.

There is an increase of reports of NIR-II based IGS using RE nanoparticles since last year. Liu and his collaborators fabricated functionalized red blood cells with RE UCNPs as a multimodal probe for NIR-II luminescence guiding precise tumor resection under an 808-nm laser irradiation and meanwhile laser activated O_2_ release to help PDT therapy for popliteal lymph node metastasis 152. In their work, it is clearly shown that NIR-II fluorescence imaging largely improves the penetration of light and exhibits lower signal-noise ratio. The penetration depth of the NIR-II fluorescence of their probe doubled in comparison of that for NIR-I fluorescence. The red blood cell and RE nanoparticles based NIR-II probe enabled the successfully NIR-II guided surgical removal of small tumor with a size of 7 mm^3^ and 3 mm^3^ (Figure 7 b,c).

All the above-mentioned reports of RE based NIR-bioimaging guided surgery concentrated on direct targeting to the tumor or immunological recognition of cancer tissues. However, Li *et al.* provided us another choice for NIR imaging based IGS with RE nanoparticles, without any targeting strategies to tumors 151. The RE nanoparticles was used for NIR-II visualization of circulatory systems instead of the tumors. Due to the moderate half-time of blood circulation, their probes are capable of monitoring vascular disorders including artery thrombosis, ischemia, and tumor angiogenesis. The cancer therapy was constructed through a blood vessel embolization surgery conducted with NIR-II navigation of femur orthotopic osteosarcoma on nude mice. In addition, the NIR-II probe is also applicable for sentinel lymph nodes imaging and sequential biopsy by tail injection.

## 6. Conclusion and Perspective

Rare earth nanoparticles have many advantages, such as high NIR luminescence efficiency, low toxicity, and good biocompatibility. They hold great promise in a wide range of applications in cancer diagnosis and treatment, and surgical navigation. However, there are only limited reports on the application of RE nanoparticles in surgical navigation at clinical level. NIR small molecular dyes and quantum dots are still the mainstream of probes for NIR fluorescence ICG. This is mainly because of the following two reasons: 1) Concerns about the safety of RE nanoparticles, including their refractoriness and toxicity of possibly released rare earth ions; 2) In order to achieve higher sensitivity and spatiotemporal resolution in IGS, smaller RE nanoparticles are required, however, the luminescence efficiency of RE nanoparticles decreases rapidly within smaller size nanoparticles 153. Whereas the nanoparticles smaller than 10 nm has no advantage against competing semiconductor quantum dots in terms of luminescence efficiency.

On the other hand, the current reports of NIR surgical navigations using lanthanide nanoparticles are mostly focused on simple animal models such as ovarian tumor metastases and unilateral thrombus on nude mice. Larger animals such as rabbits 154 and dogs 155 have not yet been employed in NIR emissive RE nanoparticles based IGS. Considering that the major advantage of using NIR emissive RE nanoparticles is to boost the penetration depth of the excitation light, it is important to verify it in larger animals with thicker tissues. Thus, it is of great urgency to develop new disease models to larger mammals which can be better mimics for human body. However, the penetration depth of NIR fluorescence of current reports are mostly no larger than 10 mm, which is obviously impractical for clinical surgery of human body. From this aspect, we shall prospect that there is still great space for the improvement of the fluorescence intensity, quantum yield, noise-to-background ratio and eventually penetration depth for the RE nanoparticles of NIR emission.

Therefore, the future development trends of RE nanoparticles in the field of NIR fluorescent IGS are proposed as follows: a) Develop degradable and metabolizable rare earth nanoparticles, where the metabolites of the nanoparticles are required to be non-toxic too; b) Further improve the luminescence efficiency of NIR, especially for small size nanoparticles, it is necessary to surpass inorganic semiconductor quantum dots (such as Ag_2_S) 156 and also improve the penetration depth of NIR fluorescence. c) Expand the unique luminescent properties such as long afterglow and time-resolved luminescence, and utilize the magnetism of rare earth elements such as gadolinium to develop multi-mode molecular imaging technology including MRI and multiple optical imaging techniques.
